# Etymologia

**DOI:** 10.3201/eid1703.ET1703

**Published:** 2011-03

## *Pseudoterranova azarasi* [sü-dō-′ter-ə-nō-və a-zär-a-sē]

From the Greek for false, Latin for earth and new, and Japanese for sea lion. First identified in 1878 as a parasite in pinnipeds by Danish scientist Harald Krabbe, who suggested the name *Ascaris decipiens*, the taxonomic designation for these nematodes changed as knowledge of the life cycles and morphologic features of members of the order Ascaridida expanded. In 1998, molecular examination found *Pseudoterranova decipiens*, long thought to be a monotype, consisted of genetically distinct sibling species. Mattiucci et al. proposed *Pseudoterranova azarasi* for 1 of the 5 sibling species, incorporating part of the name *Porrocaecum azarasi*, previously considered a synonym for *P. decipiens.*

